# Ergosterol peroxide activates Foxo3-mediated cell death signaling by inhibiting AKT and c-Myc in human hepatocellular carcinoma cells

**DOI:** 10.18632/oncotarget.8608

**Published:** 2016-04-06

**Authors:** Xiangmin Li, Qingping Wu, Ming Bu, Liming Hu, William W. Du, Chunwei Jiao, Honghui Pan, Mouna Sdiri, Nan Wu, Yizhen Xie, Burton B. Yang

**Affiliations:** ^1^ State Key Laboratory of Applied Microbiology Southern China, Guangdong Provincial Key Laboratory of Microbial Culture Collection and Application, Guangdong Institute of Microbiology, Guangzhou, PR China; ^2^ Sunnybrook Research Institute, Sunnybrook Health Sciences Centre, Toronto, Canada; ^3^ Department of Laboratory Medicine and Pathobiology, University of Toronto, Toronto, Canada; ^4^ College of Life Science and Bioengineering, Beijing University of Technology, Pingleyuan, Chaoyang, Beijing, China; ^5^ Guangdong Yuewei Edible Fungi Technology Co. Ltd, Guangzhou, China; ^6^ Institute of Medical Science, University of Toronto, Toronto, Canada

**Keywords:** ergosterol peroxide, pAKT, c-Myc, Foxo3, Puma

## Abstract

Sterols are the important active ingredients of fungal secondary metabolites to induce death of tumor cells. In our previous study, we found that ergosterol peroxide (5α, 8α-epidioxiergosta-6, 22-dien-3β-ol), purified from *Ganoderma lucidum*, induced human cancer cell death. Since the amount of purified ergosterol peroxide is not sufficient to perform *in vivo* experiments or apply clinically, we developed an approach to synthesize ergosterol peroxide chemically. After confirming the production of ergosterol peroxide, we examined the biological functions of the synthetic ergosterol peroxide. The results showed that ergosterol peroxide induced cell death and inhibited cell migration, cell cycle progression, and colony growth of human hepatocellular carcinoma cells. We further examined the mechanism associated with this effect and found that treatment with ergosterol peroxide increased the expression of Foxo3 mRNA and protein in HepG2 cells. The upstream signal proteins pAKT and c-Myc, which can inhibit Foxo3 functions, were clearly decreased in HepG2 cells treated with ergosterol peroxide. The levels of Puma and Bax, pro-apoptotic proteins, were effectively enhanced. Our results suggest that ergosterol peroxide stimulated Foxo3 activity by inhibiting pAKT and c-Myc and activating pro-apoptotic protein Puma and Bax to induce cancer cell death.

## INTRODUCTION

Hepatocellular carcinoma is one of the most common neoplasms worldwide, which frequently develops in the site of chronic hepatitis or cirrhosis [1]. It would be helpful to prevent the development of hepatocellular carcinoma in the high-risk group of patients with chronic hepatitis or cirrhosis. Natural health products have attracted extensive attention in health promotion and disease treatment including cancer [2–4]. In addition, natural product-based drug discovery is a major route leading to developing therapeutic drugs for various diseases including cancer. Medicinal mushrooms are a large group of natural product which are extensively used in health promotion and drug development [5–9]. *Ganoderma lucidum* is the most known medicinal mushroom and is regarded as the folk medicine used for prevention and treatment of various human diseases, especially cancer [10–15]. The other members of this family also possess anti-tumor activity [16, 17].

Our previous study showed that the oil fraction isolated from the Ganoderma spores was very powerful in inducing cancer cell death [18]. Further study found that the Ganoderma oil could induce death of cancer stem-like cells [11]. We purified the bioactive components and finally isolated the single molecule ergosterol peroxide from this medicinal mushroom. We found that ergosterol peroxide could stimulate cell death of a panel of cancer cells including human hepatocellular carcinoma cells HepG2 [11]. Erogosterol peroxide is a member of a class of fungal secondary metabolites of 5α, 8α-endoperoxide sterol derivatives. It can be isolated from many medicinal fungi, such as *Sarcodon aspratus, Hericium erinaceum, Armillariella mellea, lactarius hatsudake, hypsizigus marmoreus* [19–21]. It have been reported that ergosterol peroxide can inhibit tumor growth by anti-angiogenesis or cytotoxicity [11, 22]. However, the amount of ergosterol peroxide, isolated from fungi, was too little, which was not sufficient to be used clinically. In this study, we firstly developed an approach to synthesize ergosterol peroxide. After confirming the purity of the chemical, we investigated the molecular mechanisms by which the cell death of human hepatocellular carcinoma cells was induced. We found that ergosterol peroxide could reduce phosphorylated AKT (pAKT) and c-Myc expression, but could increase levels of tumor suppressor Foxo3 and activate Puma and Bax. We concluded that the activation of Foxo3 is required for ergosterol peroxide-induced cancer cell death, which is strongly associated with pro-apoptotic protein Bax and Puma.

## RESULTS

### Chemical synthesis of ergosterol peroxide

**Figure 1:**
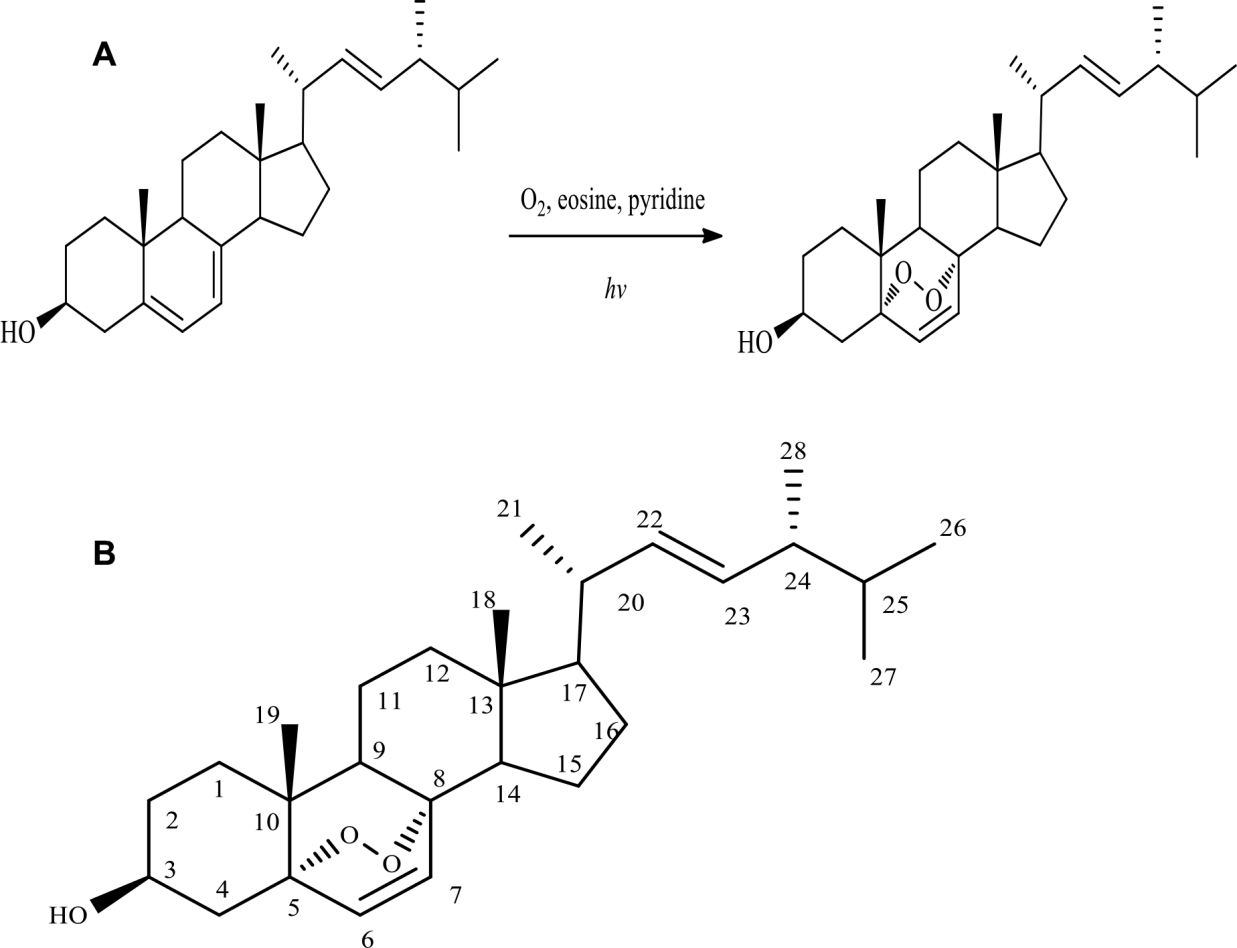
Synthesis of ergosterol peroxide (**A**) Diagram showing synthesis of ergosterol peroxide from ergosterol. (**B**) Molecular structure of ergosterol peroxide.

Using ergosterol as the starting material, we performed chemical synthesis and purification as described in the Materials and Methods. A product named Compound I was obtained. Compound I appeared to be a white crystalline needles, mp180–182°C (uncorr.). Structural analysis showed the following parameters: ESI-MS *m/z*: 451 [M+Na]^+^, 467[M+K]^+^. ^1^H NMR (400 MHz, CDCl_3_): δ 0.82 (3H, d, *J* = 6.8 Hz, H-27), 0.83 (3H, s, H-18), 0.84 (3H, d, *J* = 6.8 Hz, H-26), 0.89 (3H, s, H-19), 0.91 (3H, d, *J* = 6.9 Hz, H-28), 1.00 (3H, d, *J* = 6.4 Hz, H-21), 3.97 (1H, tt, *J* = 5.04, 11.47 Hz, H-3), 5.12 (1H, dd, *J* = 8.0, 15.2 Hz, H-22), 5.23 (1H, dd, *J* = 7.6, 15.2 Hz, H-23), 6.24 (1H, d, *J* = 8.4 Hz, H-6), 6.51 (1H, d, *J* = 8.4 Hz, H-7). ^13^C NMR (100 MHz, CDCl_3_): δ 12.9 (C-18), 17.6 (C-28), 18.2 (C-19), 19.6 (C-21), 19.9 (C-27), 20.6 (C-26), 20.9 (C-11), 23.4 (C-15), 28.6 (C-16), 30.1 (C-2), 33.1 (C-25), 34.7 (C-10), 37.0 (C-1), 37.0 (C-14), 39.3 (C-12), 39.7 (C-20), 42.8 (C-24), 44.6 (C-13), 51.1 (C-4), 51.7 (C-9), 56.2 (C-17), 66.4 (C-3), 79.4 (C-5), 82.2 (C-8), 130.7 (C-24), 132.3 (C-23), 135.2 (C-7), 135.4 (C-22). The spectral data of Compound I were consistent with ergosterol peroxide (5α, 8α-epidioxiergosta-6, 22-dien-3β-ol, EPO)^[2]^. Figure 1 showed that ergosterol peroxide was synthesized from ergosterol. Using 150 mg ergosterol, 104 mg ergosterol peroxide was obtained with a yield of 64%.

### Ergosterol peroxide inhibited viability of human hepatocellular carcinoma cells

**Figure 2:**
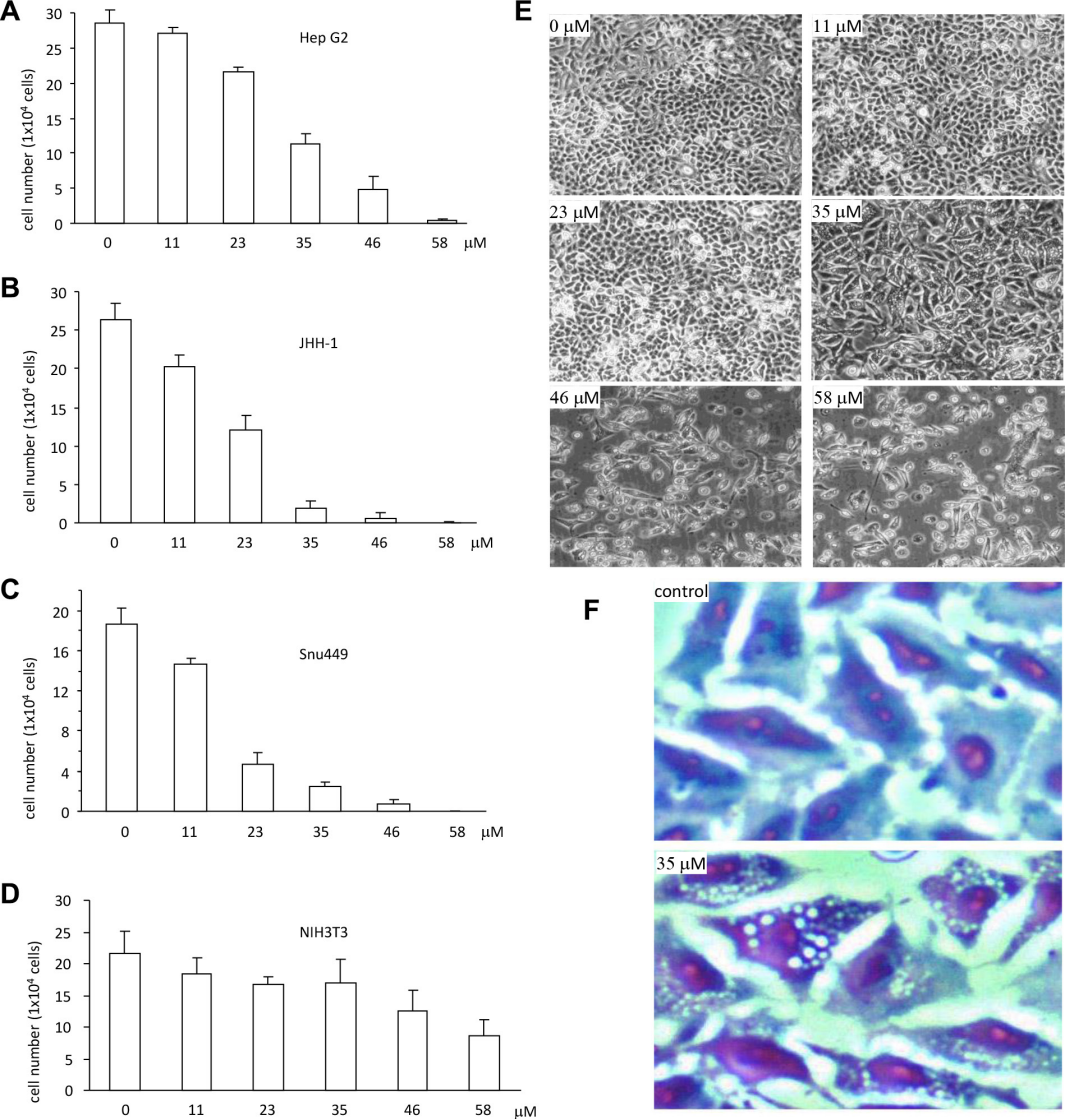
Ergosterol peroxide induced cell death of human hepatocellular carcinoma (**A–D**) Ergosterol peroxide was added to the cultures of HepG2 (A), JHH-1 (B), Snu449 (C) and NIH3T3 (D) at the concentrations of 0, 11, 23, 35, 46, 58 μM, followed by 48 h incubation. Cell viability was analyzed by trypan blue staining and cell counting. Each experiment was repeated three times. All results were from three experiments taken as mean ± SD. (**E**) HepG2 cells were treated with different concentrations of ergosterol peroxide as indicated. Typical photos of cell death were shown. (**F**) HepG2 cells were treated with ergosterol peroxide at the concentration of 35 μM for 24 h. Cells were fixed with cold methanol for 15 min and stained with the Diff-Quik-Stain kit, followed by microscopic examination.

To investigate the anticancer effect of the synthetic ergosterol peroxide, we performed cell proliferation assay followed by treating the human hepatocellular carcinoma cell lines HepG2, JHH-1 and SNU-449 with different concentrations of ergosterol peroxide. After the treatment, the cells were subjected to viability analysis stained with trypen blue. As a control, a normal mouse embryo fibroblast cell line NIH3T3 was used. We have previously shown that while Ganoderma oil induced death of a number of cancer cell lines, it had little effect on NIH3T3 cells [18]. Our experiments showed that treatment with the synthetic ergosterol peroxide inhibited viability of HepG2 cells in a dose-dependent manner (Figure 2A). We also performed similar experiments in other liver cancer cell lines JHH-1 and SNU444, as well as a non-cancer cell line NIH3T3. As shown in the Figure 2B and Figure 2C, similar results were obtained in the JHH-1 and SNU-449 cells treated with the synthetic ergosterol peroxide. Both JHH-1 and SNU-449 cells appeared to be more sensitive to ergosterol peroxide in the inhibition of cell viability relative to HepG2 cells. In the non-cancer cell line NIH3T3 fibroblasts, we detected significantly less sensitive of the cells to the synthetic ergosterol peroxide (Figure 2D). Careful examination of the cells treated with ergosterol peroxide revealed the typical vacuoles, which showed the greatest number in the cultures treated with 35 μM of ergosterol peroxide (Figure 2E). Cell staining with Diff-Quik-Stain kit revealed clear vacuoles (Figure 2F).

**Figure 3:**
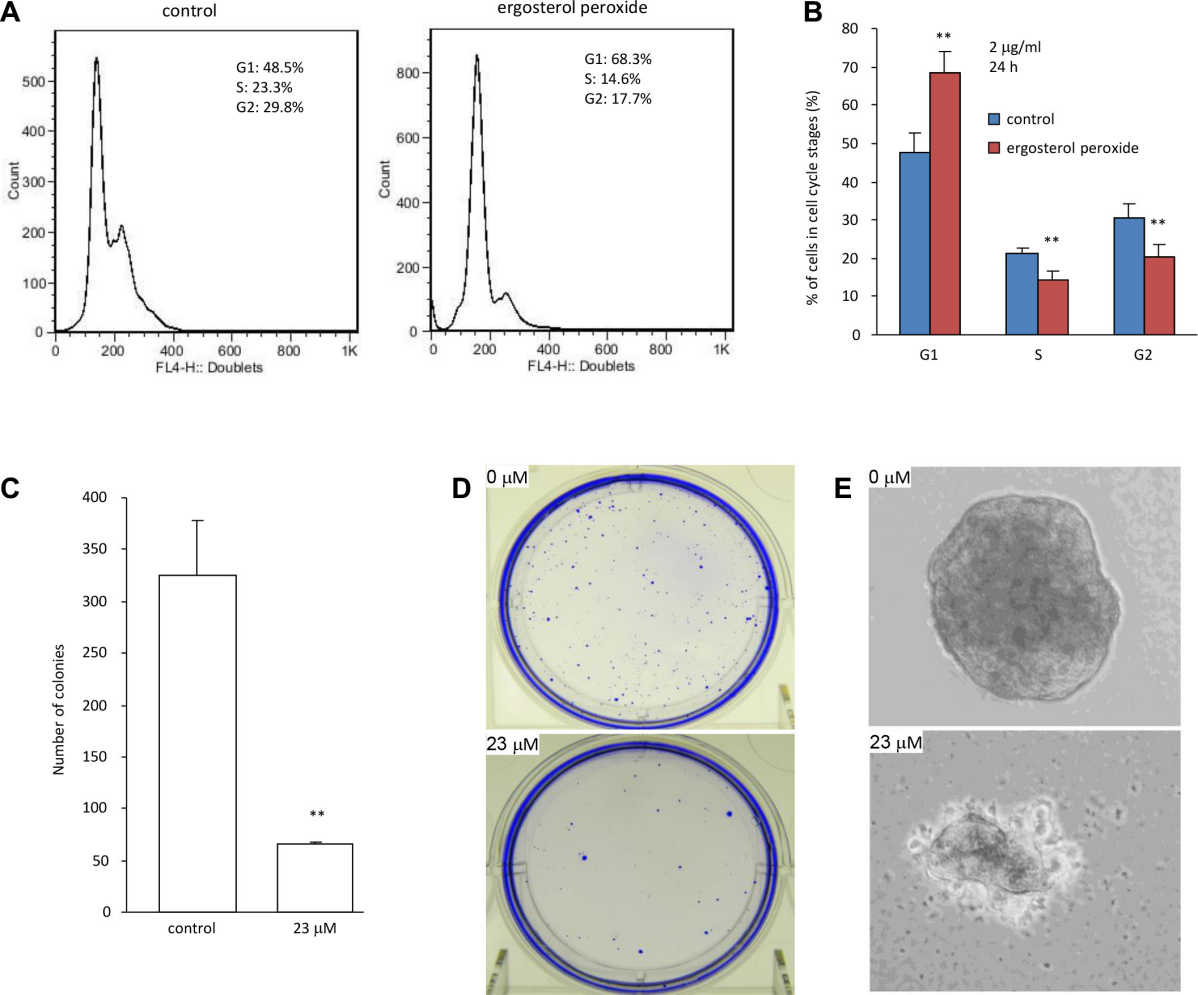
Ergosterol peroxide inhibited cell cycle progression and colony formation (**A**) Typical distribution of cell cycles treated with or without ergosterol peroxide. (**B**) Statistical analysis of cells distributed in each phase of cell cycles when the cells were treated with or without ergosterol peroxide. (**C**) Colony formation assay was performed in HepG2 cells in the presence or absence of ergosterol peroxide (23 μM). 20 days after incubation, colonies were counted. Data represent the mean ± SD of three experiments. Asterisks indicate significant differences. **p < 0.01. Error bars, SD (*n = 3*). (**D**) Culture plates containing colonies were stained with Coomassie Blue. Typical photos of the plates are shown. (**E**) Typical photos of colonies are shown.

The Effect of ergosterol peroxide on cell cycle progression was analyzed by flow cytometry. To avoid induction of cell death, HepG2 cells were treated with very low concentration of ergosterol peroxide (2 μg/ml). We detected increased number of cells in the G1 phase after the cultures were treated with ergosterol peroxide for 24 hours (Figure 3A), suggesting an inhibitory effect of the chemical on cell cycle progression. Indeed, the number of cells decreased in S-G2 phases significantly when the cells were treated with ergosterol peroxide (Figure 3B).

Since HepG2 is a non-tumorigenic cell line reported on ATCC website (http://www.atcc.org/products/all/HB-8065.aspx#characteristics), it would be difficult to show the inhibitory effect of ergosterol peroxide on tumor growth *in vivo*. We thus analyzed the effect of this chemical on colony formation *in vitro*. HepG2 cells were treated with 23 μM ergosterol peroxide followed by examination of colony formation under light microscopy. We found that HepG2 cells treated with ergosterol peroxide formed significantly lower number of colonies than the untreated cells (Figure 3C–3D). Furthermore, the sizes of the colonies appeared smaller in the cells treated with ergosterol peroxide relative to the untreated cells (Figure 3E).

**Figure 4:**
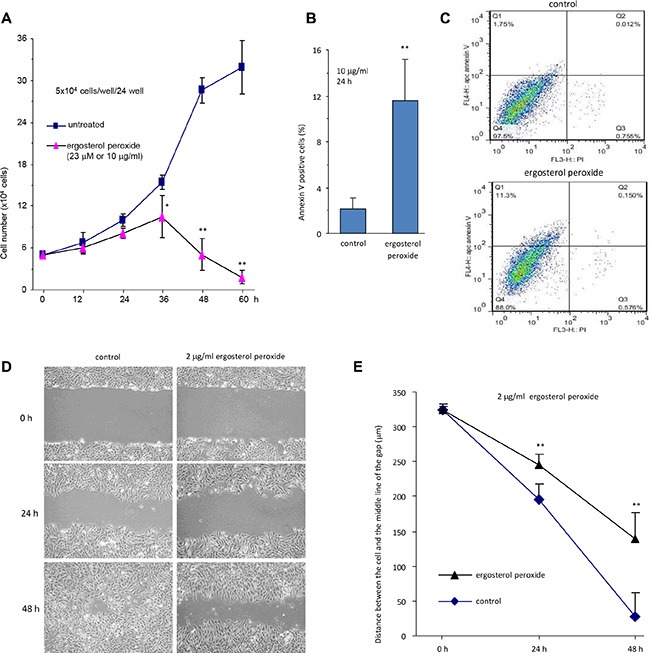
Ergosterol peroxide decreased cell survival and migration (**A**) HepG2 cells were cultured in the presence or absence of ergosterol peroxide (23 μM, 10 μg/ml) for up to 60 h. Cell viability was measured by trypan blue staining. The experiment was repeated three times. All data were taken as mean ± SD from three experiments. Asterisks indicate significant differences. *p < 0.05, **p < 0.01. Error bars, SD (*n = 3*). (**B**) HepG2 cells were treated with or without ergosterol peroxide for 24 h, followed by apoptosis analysis. Treatment with ergosterol peroxide induced cell apoptosis. Asterisks indicate significant differences. **p < 0.01. Error bars, SD (*n = 3*). (**C**) Typical captures of cell apoptosis, treated with or without ergosterol peroxide. (**D**) Cell migration was monitored using scratch assay. It was found that ergosterol peroxide-treated HepG2 cells displayed lower speeds of motility than the control cells. Typical photos of cell motility are shown. (**E**) Migration rates were measured and calculated. HepG2 cells treated with ergosterol peroxide displayed significantly lower rate of migration compared to the control cells. Asterisks indicate significant differences. **p < 0.01. Error bars, SD (*n = 3*).

We also treated the cells with relatively low concentrations of the synthetic ergosterol peroxide but longer periods of times. It was found that the number of HepG2 cells treated with 23 μM (10 μg/ml) of ergosterol peroxide started to decline after incubation for 36 hours (Figure 4A). At this concentration, HepG2 cells began to undergo apoptosis after being treated for 24 hours (Figure 4B), measured by flow cytometry (Figure 4C). We also found that incubation with ergosterol peroxide inhibited cell migration in a wound healing assay of monolayer cultures (Figure 4D). The inhibitory effect of ergosterol peroxide reached the level of significance at 24 hours (Figure 4E).

### Ergosterol peroxide regulated Foxo3 expression and nuclear localization in HepG2 cells

**Figure 5:**
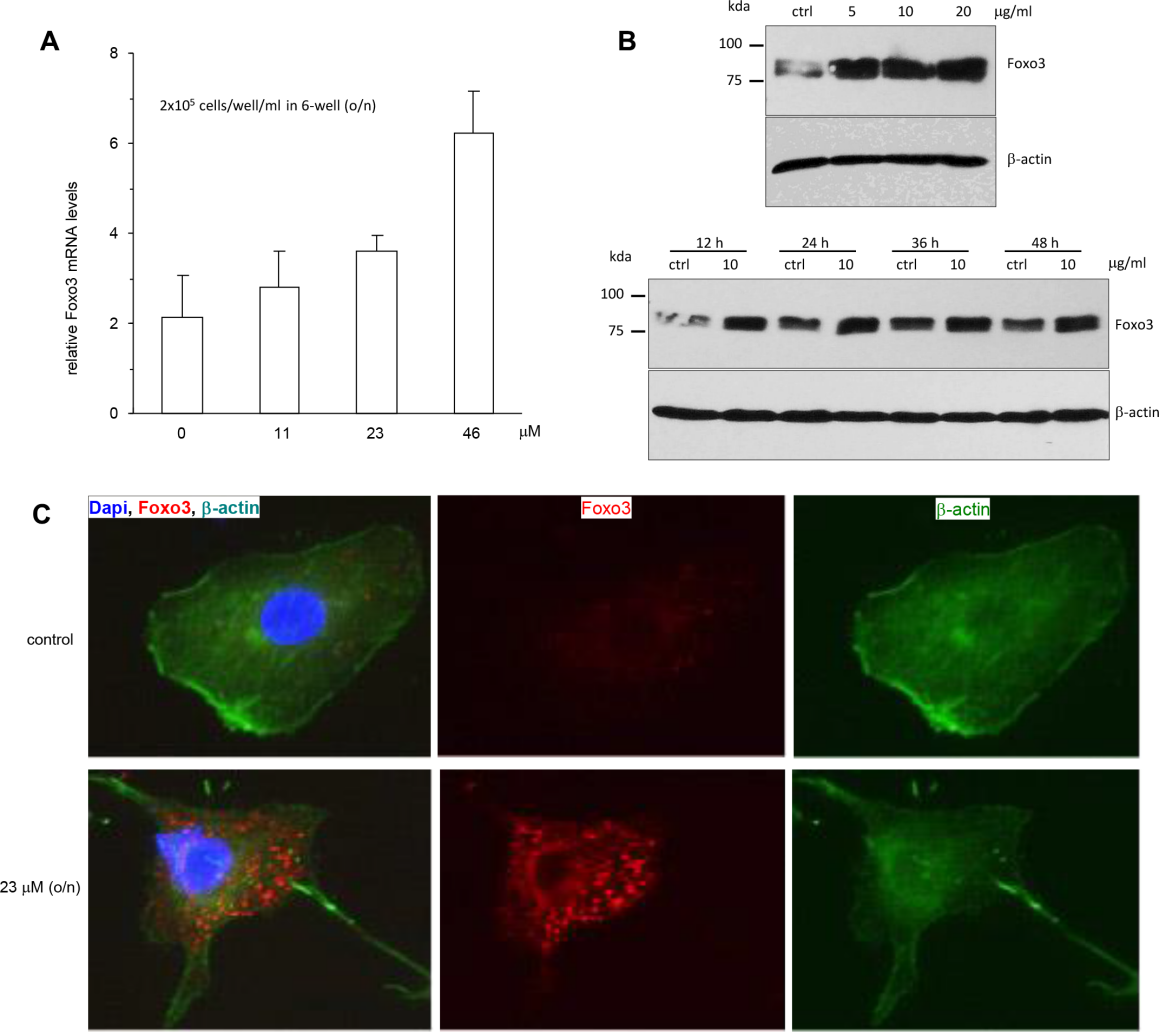
Promotion of Foxo3 expression by ergosterol peroxide treatment (**A**) HepG2 cells were treated with or without ergosterol peroxide at the concentrations (0–46 μM) for 15 h. Total RNA was prepared and subject to real-time PCR analysis. Treatment with ergosterol peroxide increased the expression of Foxo3 mRNA. (**B**) Upper, HepG2 cells were treated with or without ergosterol peroxide at the concentrations (0–20 μM) for 15 h. Cells lysates were prepared and subject to Western blot analysis probed with anti-Foxo3 antibody. Treatment with ergosterol peroxide increased expression of Foxo3 in a concentration-dependent manner. Lower, The cells were treated with or without ergosterol peroxide at 10 μM for different time points, followed by Western blotting. The promotion of Foxo3 expression was not time-dependent. (**C**) HepG2 cells were treated with or without ergosterol peroxide at 23 μM for 15 h. The cells were subject to immunostaining for Foxo3 (red), β-actin (green) for cell structure and DAPI (blue) for nuclear examination. Greater levels of Foxo3 were mainly detected in the cytoplasm of the cells treated with ergosterol peroxide.

Foxo3 is a forkhead-box O3 transcription factor that inhibits tumor cell growth and induces apoptosis by activating pro-apoptotic genes [23]. We explored the potential effect of Foxo3 in mediating ergosterol peroxide-induced cell death. HepG2 cells treated with different concentrations of ergosterol peroxide were subject to real-time PCR analysis for Foxo3 expression. The PCR results showed that treatment with ergosterol peroxide increased the mRNA expression of Foxo3 in a concentration dependent manner compared to the untreated cells (Figure 5A). Western blot analysis also displayed a concentration-dependent effect of ergosterol peroxide on Foxo3 protein levels (Figure 5B, upper). Nevertheless, the effect of ergosterol peroxide on Foxo3 protein levels appeared not to be time-dependent (Figure 5B, lower). It suggested that the effect of Foxo3 in mediating ergosterol peroxide function might be an early event in ergosterol peroxide-induced cell death. We examined subcellular localization of Foxo3 in the HepG2 cells treated with or without ergosterol peroxide by immune-fluorescence assay. It showed that the majority of Foxo3 was localized in the cytoplasm of the cells after ergosterol peroxide treatment as compared to the control (Figure 5C).

### Ergosterol peroxide decreased pAKT (Ser473) protein expression in HepG2 cells

**Figure 6:**
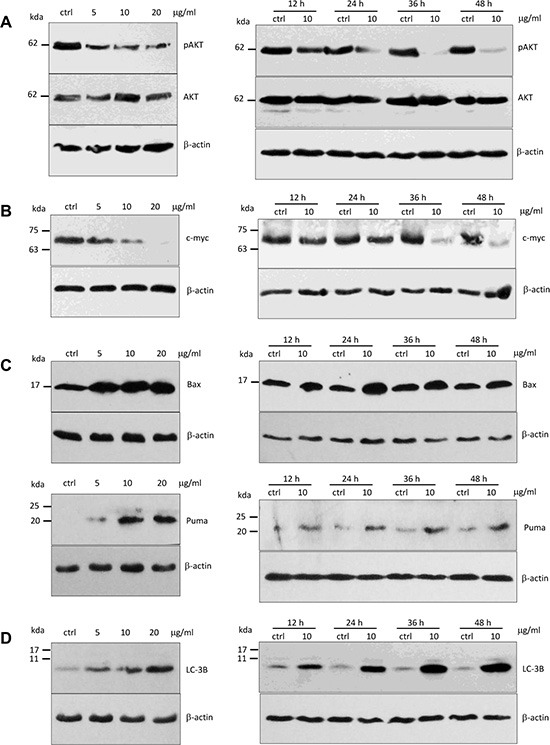
Expression of pAKT, c-Myc, Puma, Bax and LC-3B in HepG2 cells treated with ergosterol peroxide (**A**) HepG2 cells were treated with ergosterol peroxide at the concentrations and time pointed as indicated, followed by Western blotting probed with oncogenic proteins pAkt and Akt. (**B**) Treatment with ergosterol peroxide decreased c-myc levels in the concentration- and time-dependent manner. (**C**) Upper, Treatment with ergosterol peroxide increased Bax expression. Lower, Treatment with ergosterol peroxide increased Puma expression. (**D**) Treatment with ergosterol peroxide increased LC-3B levels in the concentration- and time-dependent manners.

It has been reported that Foxo3 protein shuttling between nuclear and cytoplasmic fractions was mainly regulated by the activity of Akt kinases [24]. To determine whether up-regulation and nuclear localization of Foxo3 as a consequence of ergosterol peroxide treatment was modulated by inhibiting AKT activity, we analyzed the expression of pAKT (Ser473) and AKT with Western blotting. We found that the levels of pAKT (Ser473) decreased significantly in a concentration- and time-dependent manner in the ergosterol peroxide-treated cells relative to the untreated cells (Figure 6A). However, AKT expression has no affected.

### Ergosterol peroxide decreased c-myc expression

Activation of c-myc is associated with tumor growth including DNA replication, gene transcription, protein synthesis, and cell proliferation [25]. To determine whether ergosterol peroxide also reduced c-myc protein expression in HepG2 cells, we examined c-myc protein levels by Western bolt analysis. We found that the c-myc protein levels significantly decreased in concentration- and time-dependent manners (Figure 6B). When the cells were treated with ergosterol peroxide at the concentration of 20 μg/ml, the level of c-myc was undetectable.

### Ergosterol peroxide up-regulated Bax and Puma protein expression

It has been reported that the genes of Bcl-2 family are important pro-apoptotic targets of Foxo3 protein, including *BAX and PUMA* [23, 26]. We examined *BAX and PUMA* expression in the cells treated with ergosterol peroxide. Western blot analysis showed that ergosterol peroxide treatment increased the expression of *BAX and PUMA* (Figure 6C). These results suggested that Foxo3 could mediate the effect of ergosterol peroxide by activating *BAX* and *PUMA* leading to cancer cell death.

Since expression of LC-3B is an indication of autophagy and severe autophagy can induce cell death, we examined the effect of ergosterol peroxide on LC-3B levels and found that ergosterol peroxide could remarkably induced the expression of LC-3B (Figure 6D), suggesting a stimulation of HepG2 autophagy by ergosterol peroxide.

## DISCUSSION

Ergosterol peroxide is a natural products isolated from many species of medicinal fungi. It has been shown to possess many biological activities including anti-oxidation, anti-inflammation, and anti-tumor cell growth [11, 19]. Rhee and co-worker have reported that ergosterol peroxide inhibited the signaling pathway proteins JAK2/STAT3, which are important in angiogenesis, and exerted anti-tumor activity in multiple myeloma U266 cells [22]. In our previous study, we found that ergosterol peroxide, purified from medicinal mushroom *Ganoderma lucidum*, induced human cancer cell death [11]. In this study, we firstly synthesized ergosterol peroxide from ergosterol and used the synthetic chemical to study its anti-tumor mechanism.

It have been reported that *Ganoderma lucidum* posses hepatoprotective activity and can induce HepG2 cell death [27]. In the present study, we found that the chemically synthesized ergosterol peroxide played the similar roles in inhibiting cell cycle progression and migration of hepatocellular carcinoma cells. The ergosterol peroxide induced cell death was further confirmed by colony formation assay. Our results demonstrated that the effects of synthetic ergosterol peroxide on tumor cells are similar to those of the naturally purified chemical. Our results also suggested that the synthetic ergosterol peroxide might be used for preclinical test and clinical application.

We explored the molecular mechanism of ergosterol peroxide in antitumor effects. Our results showed that Foxo3 protein level was up-regulated in HepG2 cells in response to ergosterol peroxide treatment, since Foxo3 functions as a tumor suppressor [28–31]. In addition, Foxo3 was translocated to nucleus. It is known that up-regulation and nuclear localization of Foxo3 activates its downstream target genes, such as Bim, Bax and Puma, leading to cancer cell death [23, 26]. Bax is a multidomain pro-apoptotic Bcl-2 family protein, which can be activated by BH3-only molecules such as Bim, Bid and Puma to trigger cell death [25, 26]. BCL2 family members can form hetero- or homodimers and are involved in a variety of cellular activities. Bax activation can be stimulated by various factors, including hydrogen peroxide and mitochondrial membrane remodeling. It can also be activated by binding to Bcl-2 or other proteins. Our results showed that Bax and Puma were up-regulated in HepG2 cell subjected to ergosterol peroxide treatment. Based on these results, we proposed a new mechanism by which Foxo3 induces cell death in response to ergosterol peroxide treatment through activation of Bax by BH3-only molecule Puma.

The activation of Foxo3 was inhibited via phosphorylation by Akt activation [23, 24]. The proto-oncogene Akt is activated via phosphorylation at Thr308 and Ser473 in most types of human cancers including hepatocarcinoma [24, 32]. The activated Akt can then phosphorylate Foxo3 in the nucleus, which is degraded to block it in targeting its downstream genes and triggering cell death pathway [23, 24]. Our results showed that pAkt protein was reduced in response to ergosterol peroxide treatment. Thus, it is important that the activation of Akt was repressed in order to promote up-regulation of Foxo3 and to facilitate its localization to nucleus in HepG2 cells after ergosterol peroxide treatment.

We also found that c-Myc protein decreased in the concentration- and time-dependent manner in HepG2 cells after ergosterol peroxide treatment. It have been reported that c-Myc can compete with Foxo3 to bind the apoptosis-promoting proteins that expressed by downstream target genes of Foxo3, for example Puma, to inhibit the cell death-inducing activity of Foxo3 [25]. Therefore, we proposed that ergosterol peroxide treatment induced cancer cell death via enhancing the activation of Foxo3 by repressing c-Myc to promote pro-apoptotic genes Bax and Puma expression.

In summary, we report an approach to synthesize ergosterol peroxide and investigated the anti-tumor activity. Using the synthetic molecule, it allowed us to examine the molecular mechanism in anti-cancer cell activity. We have thus shown that Foxo3 played an important role in mediating cell death in response to ergosterol peroxide. This may be largely due to the key role of Foxo3 in mediating a variety of signaling pathways. Our results suggest that ergosterol peroxide can inhibit oncogenic AKT and c-Myc to activate the expression of Foxo3, which in turn activates the downstream apoptosis promoting gene Puma and Bax to initiate cancer cell apoptosis pathways. This suggests that Foxo3 may be an important target in drug screening for cancer intervention.

## MATERIALS AND METHODS

### Synthesis of ergosterol peroxide

A solution of ergosterol (150 mg) and eosine (1 mg) in pyridine (10 mL) was prepared in a quartz tube. The tube kept in a water-cooled bath was saturated with oxygen. The solution, vigorously stirred by the gas bubbling, was irradiated with light from a 220 V 200 W iodine tungsten lamp placed at a distance of 15 cm. The oxygen was kept bubbling during the irradiation for 1 h. The irradiated solution was poured into ice water and extracted with ethyl acetate. The combined organic extracts were washed with brine and dried with MgSO_4_. Then the solid residue was dissolved in minimum volume of ethyl acetate and adsorbed on silica gel. This silica gel was added to the top of a silica chromatography column and eluted with hexane-ethyl acetate (3:1). A product was obtained named Compound I.

### Structural analysis of compound I

The melting point of Compound I was measured by a XT4A electro-thermal apparatus equipped with a microscope and was uncorrected. ^1^H NMR and ^13^C NMR spectra were recorded on an AV 400M Bruker spectrometer. Chemical shifts were measured in CDCl_3_ with tetramethyl-sislane (TMS) as internal reference, and chemical shifts (δ) were reported in parts per million (ppm). The MS spectra (ESI) were recorded on a Bruker Esquire 6000 mass spectrometer.

### Cell proliferation assay

The effect of Compound I on cell proliferation was evaluated by trypan blue staining assay. Human hepatocellular carcinoma cells HepG2, JHH-1, and SNU449 were used in the study. The cells were cultured in DMEM or RPMI1640 supplemented with 10% FBS, 100 U/ml penicillin/streptomycin at 37°C in an incubator containing 5% CO_2_. Cells (1 × 10^5^cells/ml) were seeded into 24-well plates with 500 μL complete culture medium. Four hours after cell inoculation, Compound I was added to the cell cultures. The cells were incubated for various periods and analyzed by trypan blue staining to analyze rates of cell proliferation as described [33, 34].

### Cell migration assay

Migration assay was performed by wound scratch test as described [35, 36]. Briefly, 3 × 10^5^ cells were seeded in each well of 6-well plates and incubated with or without ergosterol peroxide. The cells were scratched using pipette tips. The cultures were washed with PBS to remove cell debris. Ergosterol peroxide was added to the cultures at a concentration of 2 μg/ml. Cellular motility was monitored and photographed. Microscope images of the scratched cultures were captured at the beginning and at different intervals later. Distance travelled from the initial scratch site was measured and the migration distance was quantified.

### Flow cytometry

Cell apoptosis was analyzed by Annexin V apoptosis detection kit APC (eBioscience, Inc., USA) as described [37, 38]. In brief, the cultured cells were washed, trypsinized, collected, and resuspended, followed by incubation with Annexin V (APC) in the dark at room temperature for 15 min. The cells were then washed and resuspended in 400 μl binding buffer, to which 5 μl of Propidium Iodide solution were added. The cells were then analyzed by flow cytometry (FACS Calibur, Becton, Dickinson and Company. USA). The cells showing Annexin V-positive and PI-negative were counted as apoptotic, whereas PI-positive staining was counted as necrotic cells.

For cell cycle analysis, cells in the logarithmic phase were harvested and washed twice in PBS. The cells were resuspended in cold PBS and incubated in ice-cold 70% ethanol for 3 h. After centrifugation at 1,500 rpm for 10 min, the cells were resuspended in propidium iodide (PI) master mix (40 mg/ml PI and 100 mg/ml RNase in PBS) at a density of 5 × 10^5^ cells/ml and incubated at 37°C for 30 min before analysis with flow cytometry as described [39, 40].

### Colony formation

Colony formation assay was performed as previously described [41, 42]. Briefly, HepG2 cells (500 cells/well) were mixed with 0.25% low melting agarose gel containing ergosterol peroxide at 23 μM and seeded in 6-well plates, which had been pre-coated with 0.66% regular agarose gel. The plates were maintained in a CO_2_ incubator. Two weeks after cell inoculation, colonies were counted, fixed, stained, and photographed under a microscope.

### Real-time PCR

Total RNA was extracted using Total RNA Mini kit (Frogga Bio Inc, Canada) as described [16, 43]. The RNA concentration was measured by NanoDrop 2000c UV-VIS Spectrophotometer under 260 nm. Equal amount of RNA was used for reverse-transcription to obtain cDNA for real-time PCR amplification. The cDNA was diluted as template and performed in a real-time thermal cycler using a SYBR Green PCR Kit as follows: 40 cycles of denaturation at 95°C for 5 sec, annealing at 59°C for 30 sec, followed by an elongation step at 72°C for 30 sec. The Foxo3 was analyzed using U6 as an internal control for product normalization. All tests were performed in triplicate.

### Western blot

HepG2 cells were seeded into 6-well tissue culture plates and treated with or without ergosterol peroxide at different concentrations as indicated in each figure. After the treatment for different periods of time, the cells were lysed by lysis buffer supplemented with protease inhibitor cocktail. The cells were placed on ice for 30 min and the lysates were centrifuged at 13,000 rpm at 4°C for 15 min to obtain supernatant. Protein concentrations were measured by Bio-Rad protein assay (Bio-Rad Laboratories, Inc, USA). Samples with the equal concentrations of proteins were resolved on SDS-PAGE, and the proteins were then transferred onto nitrocellulose membranes. The membranes were incubated in blocking buffer with 5% milk, and then stained with primary antibodies against phospho-AKT(S473), AKT, Foxo3, c-myc, Puma and Bax (1:1000 dilution) at 4°C overnight. After washing, the membranes were incubated with goat anti-rabbit or goat anti-mouse HRP conjugated secondary antibodies at room temperature for 2 h. After being extensively washed, the membranes were developed with ECL kits (Millipore, USA) and visualized using the Kodak autoradiography films. The same membranes were reprobed for β-actin levels to confirm equal loading.

### Immunofluorescence

Cells were seeded on chamber slides (BD Biosciences, USA) and treated with or without ergosterol peroxide at 10 μg/ml at 37°C for 12 h. The slides were fixed with 4% formaldehyde for 15 min, and blocked with 10% goat serum in PBS. The slides were then incubated with anti-Foxo3 antibody at 4°C overnight. After brief rinse with PBS, the slides were incubated with Alexa Fluor 555 goat anti-rabbit HRP conjugated secondary antibodies at room temperature for 2 h in a container avoiding light. The slides were rinsed in PBS and incubated with phalloidin (Alexa Fluor 488) for F-actin staining under the dark at room temperature for 30 min. The same slides were then rinsed with PBS and incubated with DAPI for nuclear staining under the dark at room temperature for 30 min. After being washed with PBS and mounted with mounting medium (Dako, USA), the slides were subject to image examination under a fluorescence microscope (Carl Zeiss, Germany).

### Statistical analysis

All experiments were performed in triplicate or as indicated in the experiments and numerical data were subject to independent sample *t* test. The levels of significance were set at *p < 0.05 and **p < 0.01.

## References

[1] Thorgeirsson SS, Grisham JW (2002). Molecular pathogenesis of human hepatocellular carcinoma. Nat Genet.

[2] Liu T, Men Q, Wu G, Yu C, Huang Z, Liu X, Li W (2015). Tetrandrine induces autophagy and differentiation by activating ROS and Notch1 signaling in leukemia cells. Oncotarget.

[3] Wu J, Wu Y, Yang BB (2002). Anticancer activity of Hemsleya amabilis extract. Life Sci.

[4] Wong VK, Chiu P, Chung SS, Chow LM, Zhao YZ, Yang BB, Ko BC (2005). Pseudolaric acid B, a novel microtubule-destabilizing agent that circumvents multidrug resistance phenotype and exhibits antitumor activity *in vivo*. Clin Cancer Res.

[5] Gao G, Chen L, Li J, Zhang D, Fang Y, Huang H, Chen X, Huang C (2014). Isorhapontigenin (ISO) inhibited cell transformation by inducing G0/G1 phase arrest via increasing MKP-1 mRNA Stability. Oncotarget.

[6] Pozarowski P, Halicka DH, Darzynkiewicz Z (2003). Cell cycle effects and caspase-dependent and independent death of HL-60 and Jurkat cells treated with the inhibitor of NF-kappaB parthenolide. Cell Cycle.

[7] Ryu E, Son M, Lee M, Lee K, Cho JY, Cho S, Lee SK, Lee YM, Cho H, Sung GH, Kang H (2014). Cordycepin is a novel chemical suppressor of Epstein-Barr virus replication. Oncoscience.

[8] Huang TT, Wu SP, Chong KY, Ojcius DM, Ko YF, Wu YH, Wu CY, Lu CC, Martel J, Young JD, Lai HC (2014). The medicinal fungus Antrodia cinnamomea suppresses inflammation by inhibiting the NLRP3 inflammasome. J Ethnopharmacol.

[9] Axelrod M, Gordon VL, Conaway M, Tarcsafalvi A, Neitzke DJ, Gioeli D, Weber MJ (2013). Combinatorial drug screening identifies compensatory pathway interactions and adaptive resistance mechanisms. Oncotarget.

[10] Liu DL, Li YJ, Yang DH, Wang CR, Xu J, Yao N, Zhang XQ, Chen ZS, Ye WC, Zhang DM (2015). Ganoderma lucidum derived ganoderenic acid B reverses ABCB1-mediated multidrug resistance in HepG2/ADM cells. Int J Oncol.

[11] Wu QP, Xie YZ, Deng Z, Li XM, Yang W, Jiao CW, Fang L, Li SZ, Pan HH, Yee AJ, Lee DY, Li C, Zhang Z (2012). Ergosterol peroxide isolated from Ganoderma lucidum abolishes microRNA miR-378-mediated tumor cells on chemoresistance. PLoS One.

[12] Sliva D, Loganathan J, Jiang J, Jedinak A, Lamb JG, Terry C, Baldridge LA, Adamec J, Sandusky GE, Dudhgaonkar S (2012). Mushroom Ganoderma lucidum prevents colitis-associated carcinogenesis in mice. PLoS One.

[13] Zhao S, Ye G, Fu G, Cheng JX, Yang BB, Peng C (2011). Ganoderma lucidum exerts anti-tumor effects on ovarian cancer cells and enhances their sensitivity to cisplatin. Int J Oncol.

[14] Lin ZB (2001). [The integrated studies on Ganoderma lucidum Kayst. Guided by Traditional Chinese medical theories]. Zhongguo Zhong Xi Yi Jie He Za Zhi.

[15] Xie YZ LS, Yee A, La Pierre DP, Deng Z, Lee DY, Wu QP, Chen Q, Li C, Zhang Z, Guo J, Jiang Z, Yang BB (2006). Ganoderma lucidum inhibits tumour cell proliferation and induces tumour cell death. Enzyme & Microbial Technol.

[16] Pan H, Han Y, Huang J, Yu X, Jiao C, Yang X, Dhaliwal P, Xie Y, Yang BB (2015). Purification and identification of a polysaccharide from medicinal mushroom Amauroderma rude with immunomodulatory activity and inhibitory effect on tumor growth. Oncotarget.

[17] Li X, Wu Q, Xie Y, Ding Y, Du WW, Sdiri M, Yang BB (2015). Ergosterol purified from medicinal mushroom Amauroderma rude inhibits cancer growth *in vitro* and *in vivo* by up-regulating multiple tumor suppressors. Oncotarget.

[18] LaPierre DP, Lee DY, Li SZ, Xie YZ, Zhong L, Sheng W, Deng Z, Yang BB (2007). The ability of versican to simultaneously cause apoptotic resistance and sensitivity. Cancer Res.

[19] Takei T, Yoshida M, Ohnishi-Kameyama M, Kobori M (2005). Ergosterol peroxide, an apoptosis-inducing component isolated from Sarcodon aspratus (Berk.) S. Ito. Biosci Biotechnol Biochem.

[20] Kim DH, Jung SJ, Chung IS, Lee YH, Kim DK, Kim SH, Kwon BM, Jeong TS, Park MH, Seoung NS, Baek NI (2005). Ergosterol peroxide from flowers of Erigeron annuus L. as an anti-atherosclerosis agent. Arch Pharm Res.

[21] Sgarbi DB, da Silva AJ, Carlos IZ, Silva CL, Angluster J, Alviano CS (1997). Isolation of ergosterol peroxide and its reversion to ergosterol in the pathogenic fungus Sporothrix schenckii. Mycopathologia.

[22] Rhee YH, Jeong SJ, Lee HJ, Koh W, Jung JH, Kim SH, Sung-Hoon K (2012). Inhibition of STAT3 signaling and induction of SHP1 mediate antiangiogenic and antitumor activities of ergosterol peroxide in U266 multiple myeloma cells. BMC Cancer.

[23] Lam EW, Brosens JJ, Gomes AR, Koo CY (2013). Forkhead box proteins: tuning forks for transcriptional harmony. Nat Rev Cancer.

[24] Manning BD, Cantley LC (2007). AKT/PKB signaling: navigating downstream. Cell.

[25] Amente S, Zhang J, Lavadera ML, Lania L, Avvedimento EV, Majello B (2011). Myc and PI3K/AKT signaling cooperatively repress FOXO3a-dependent PUMA and GADD45a gene expression. Nucleic Acids Res.

[26] Ren D, Tu HC, Kim H, Wang GX, Bean GR, Takeuchi O, Jeffers JR, Zambetti GP, Hsieh JJ, Cheng EH (2010). BID, BIM, and PUMA are essential for activation of the BAX- and BAK-dependent cell death program. Science.

[27] Li N, Hu YL, He CX, Hu CJ, Zhou J, Tang GP, Gao JQ (2010). Preparation, characterisation and anti-tumour activity of Ganoderma lucidum polysaccharide nanoparticles. J Pharm Pharmacol.

[28] Zhou Y, Liang C, Xue F, Chen W, Zhi X, Feng X, Bai X, Liang T (2015). Salinomycin decreases doxorubicin resistance in hepatocellular carcinoma cells by inhibiting the beta-catenin/TCF complex association via FOXO3a activation. Oncotarget.

[29] Park SH, Jang KY, Kim MJ, Yoon S, Jo Y, Kwon SM, Kim KM, Kwon KS, Kim CY, Woo HG (2015). Tumor suppressive effect of PARP1 and FOXO3A in gastric cancers and its clinical implications. Oncotarget.

[30] Cheng CW, Chen PM, Hsieh YH, Weng CC, Chang CW, Yao CC, Hu LY, Wu PE, Shen CY (2015). Foxo3a-mediated overexpression of microRNA-622 suppresses tumor metastasis by repressing hypoxia-inducible factor-1alpha in ERK-responsive lung cancer. Oncotarget.

[31] Yang W, Du WW, Li X, Yee AJ, Yang BB (2015). Foxo3 activity promoted by non-coding effects of circular RNA and Foxo3 pseudogene in the inhibition of tumor growth and angiogenesis. Oncogene.

[32] Simioni C, Martelli AM, Cani A, Cetin-Atalay R, McCubrey JA, Capitani S, Neri LM (2013). The AKT inhibitor MK-2206 is cytotoxic in hepatocarcinoma cells displaying hyperphosphorylated AKT-1 and synergizes with conventional chemotherapy. Oncotarget.

[33] Wu Y, Zhang Y, Cao L, Chen L, Lee V, Zheng PS, Kiani C, Adams ME, Ang LC, Paiwand F (2001). Identification of the motif in versican G3 domain that plays a dominant-negative effect on astrocytoma cell proliferation through inhibiting versican secretion and binding. J Biol Chem.

[34] Cao L, Yao Y, Lee V, Kiani C, Spaner D, Lin Z, Zhang Y, Adams ME, Yang BB (2000). Epidermal growth factor induces cell cycle arrest and apoptosis of squamous carcinoma cells through reduction of cell adhesion. J Cell Biochem.

[35] Rutnam ZJ, Du WW, Yang W, Yang X, Yang BB (2014). The pseudogene TUSC2P promotes TUSC2 function by binding multiple microRNAs. Nat Commun.

[36] Luo L, Ye G, Nadeem L, Fu G, Yang BB, Honarparvar E, Dunk C, Lye S, Peng C (2012). MicroRNA-378a-5p promotes trophoblast cell survival, migration and invasion by targeting Nodal. J Cell Sci.

[37] Fang L, Li H, Wang L, Hu J, Jin T, Wang J, Yang BB (2014). MicroRNA-17-5p promotes chemotherapeutic drug resistance and tumour metastasis of colorectal cancer by repressing PTEN expression. Oncotarget.

[38] Li H, Gupta S, Du WW, Yang BB (2014). MicroRNA-17 inhibits tumor growth by stimulating T-cell mediated host immune response. Oncoscience.

[39] He J, Wu J, Xu N, Xie W, Li M, Li J, Jiang Y, Yang BB, Zhang Y (2013). MiR-210 disturbs mitotic progression through regulating a group of mitosis-related genes. Nucleic Acids Res.

[40] Du WW, Yang W, Liu E, Yang Z, Dhaliwal P, Yang BB (2016). Foxo3 circular RNA retards cell cycle progression via forming ternary complexes with p21 and CDK2. Nucleic acids research.

[41] Li H, Yang BB (2012). Stress response of glioblastoma cells mediated by miR-17-5p targeting PTEN and the passenger strand miR-17-3p targeting MDM2. Oncotarget.

[42] Siragam V, Rutnam ZJ, Yang W, Fang L, Luo L, Yang X, Li M, Deng Z, Qian J, Peng C, Yang BB (2012). MicroRNA miR-98 inhibits tumor angiogenesis and invasion by targeting activin receptor-like kinase-4 and matrix metalloproteinase-11. Oncotarget.

[43] Fang L, Du WW, Yang W, Rutnam ZJ, Peng C, Li H, O'Malley YQ, Askeland RW, Sugg S, Liu M (2012). MiR-93 enhances angiogenesis and metastasis by targeting LATS2. Cell Cycle.

